# Preliminary evaluation of the online course “I Care” targeting eating disorder knowledge and attitudes among sports coaches and fitness instructors

**DOI:** 10.1186/s40337-022-00663-1

**Published:** 2022-09-29

**Authors:** Sofia Selenius, Andreas Birgegård, Emma Forsén Mantilla

**Affiliations:** 1 Frisk & Fri-Riksföreningen mot ätstörningar, The National Eating Disorders Patient and Advocacy Organization, Inedalsgatan 5, 11233 Stockholm, Sweden; 2 Department of Medical Epidemiology and Biostatistics (MEB), Karolinska Institutet, Nobels väg 12A, 171 77 Stockholm, Sweden

**Keywords:** Eating disorders, Compulsive exercise, Coaches, Trainers, Fitness instructors, Confidence, Knowledge, Education

## Abstract

**Background:**

Fitness instructors, coaches and trainers are often looked up to and seen as role-models within their sporting community. Since problematic exercise is a common symptom of eating disorders, they are also highly likely to meet individuals at risk for developing eating disorders within their profession. Thus, educating coaches about how to promote healthy ideals within their sports/training context and equipping them with knowledge to be able to detect and approach individuals at risk, is of great importance.

**Method:**

We studied the pre-to-post effects of the I Care online psychoeducational intervention in 150 coaches, trainers, and fitness instructors. Variables included the Compulsive Exercise Test, as well as knowledge of eating disorders, and confidence and awareness regarding approaching, talking to, and referring individuals whose observed behavior raised concerns.

**Results:**

Results showed significant and large improvements in coaches’ confidence and knowledge regarding warning signs and how to approach an at-risk person, and a decrease in their perceived ability to recognize eating disorders by sight, e.g., insight about EDs not being recognizable just by looking at a person. Also, significant positive change in attitudes about their own rule-driven exercise behavior and lack of enjoyment of exercise was observed.

**Conclusions:**

Although the lack of a control group compels caution, the study suggests that I Care may have positive effects in terms of a “declaration of intent” toward more positive role-modeling and interactions with clients.

**Supplementary Information:**

The online version contains supplementary material available at 10.1186/s40337-022-00663-1.

## Plain English summary

Fitness instructors, coaches and trainers are often looked up to within their sporting community. They are also likely to meet individuals at risk for developing eating disorders, since excessive or compulsive exercise is a common symptom of these disorders. Coaches therefore need to know more about how to promote healthy ideals and how to detect and approach individuals at risk of developing an eating disorder.

We studied if a brief online education called I Care could improve personal compulsive exercise tendencies, knowledge about eating disorders, and readiness to approach individuals seemingly at risk, in 150 coaches, trainers, and fitness instructors.

We found that coaches felt more confident and knowledgeable about warning signs and how to approach a person at risk after completing I Care. They also showed increased insight about EDs not being recognizable just by looking at a person and their attitudes about their own exercise behavior changed in a positive way.

Although the lack of a comparison group makes conclusions tentative, I Care may have positive effects in terms of a “declaration of intent” toward more positive role-modeling and interactions with clients.

**Supplementary Information:**

The online version contains supplementary material available at 10.1186/s40337-022-00663-1.

## Introduction

Eating disorders (ED) are serious psychiatric illnesses [1] that can be described as a persistent disturbance of eating related behaviors that result in the altered consumption or absorption of food that impairs physical health or psychosocial functioning [1], and it can affect people of all genders, ages, ethnicities, sexual orientations, and religions. EDs and disordered eating among male and female athletes are more common compared with non-athletes, especially in aesthetic, weight-class, and gravitational sports [2]. The prevalence of disordered eating and ED among athletes varies in studies from 0 to 19% in males and 6–45% in females [3] but it has been noted that athletes may under-report eating problems more than others [4, 5]. In a Swedish study [6], EDs, together with depressive disorders and trauma- and stress-related disorders were the most common mental health problems among athletes.

EDs negatively affect both health and performance by causing electrolyte disturbance, dehydration, low energy availability, increased risk of illness and injury, and reduced training capacity, endurance, and strength [7]. EDs are also associated with adverse psychological consequences such as negative self-evaluation, body dissatisfaction, increased perfectionism, and social isolation.

These consequences, both medical and psychological, may be decreased by early identification and its possible positive impact on treatment [8]. Therefore, appropriate prevention, identification, and management approaches within sporting environments are essential. The American Academy of Pediatrics, the International Olympic Committee (IOC) Medical Commission, and the American College of Sports Medicine (ACSM) have recommended coaches to be provided with knowledge and problem-solving skills to better prevent, detect, and manage extreme dieting, EDs, and energy deficiency [2].

Compulsive exercise (CE) is a common component in the development and maintenance of EDs and is often observed in people with EDs [9–11]. It can be described as a craving for physical training, resulting in uncontrollable rule-driven exercise behavior with harmful consequences [12]. Sports coaches can play an important role by identifying early signs and symptoms of EDs (e.g., CE), directing athletes to professional help, and preventing the development of EDs [3, 13, 14]. Although coaches see their role as including the identification of and help with EDs, like talking to athletes and offering support, few feel confident in identifying EDs or talking to athletes at risk [14, 15]. Preventive interventions that provide coaches with more knowledge about EDs in general, early signs, and management approaches have shown positive effects on the coaches’ recognition and management of EDs, resulting in coaches being more likely to talk about EDs with their athletes [16]. Positive effects of including coaches in preventive efforts was emphasized also in a review by Bar, Cassin, and Dionne [17]. In another review, the most effective preventive interventions for EDs among coaches included education about attitudes, beliefs and behaviours associated with EDs, early detection of symptoms, and destigmatizing the discussion around EDs [18].

“I Care” is a cost-effective and easily accessible online course aiming to increase coaches’ knowledge about EDs and encourage reflection about attitudes and communication around exercise, diet, body weight, and shape. It contains key features of interventions found to be effective previously, such as an interactive format, focus on attitudes and behaviors as well as early symptoms, and concrete information on how to approach and refer people that may be at risk for ED. Our aim was to perform a preliminary investigation into whether “I Care” had a positive effect on attitudes, communication, and perceived knowledge about EDs. We also aimed to investigate whether the course affected participants’ own exercise behavior.

## Methods

### Study design

This was an exploratory interventional study with a pre-post design. Data was collected between August 2020 and May 2021. All participants provided written consent.

### Participants

Eligible participants were adult individuals (18 yrs and older) working professionally as coaches/trainers/instructors (henceforth only referred to as “coaches”) within sports and fitness and willing to go through the I Care program. The study was advertised via sports organizations that offer their staff the I Care program and via the platform “Tyngre”, a community for professionals within fitness and sports. Individuals who participated in the study received the I Care program for free (a value of approx. 35€). In total, 365 individuals who could be correctly identified based on their personal identification number consented to the study and commenced to complete the pre-measure. Out of these, 109 did not complete the post-measure and 90 were lacking essential data from either pre- or post-measure and were thus excluded (except that the 109 with missing POST data were used in attrition analyses, see below). In addition, we excluded 16 individuals as they did not complete I Care within an appropriate timespan; we applied a minimum time of 10 min to ensure that analyzed participants had taken the course seriously (mean time was 4.5 days). The final data set comprised 150 participants, 121 (81%) females and 29 males (mean age = 31.3, *SD* = 9.36).

### The intervention: I Care

The I Care program consists of three parts. The first part focuses on ED warning signs, symptoms, and common myths. The second part focuses on healthy leadership; acknowledging the important role a coach has as a role model for healthy values linked to body ideals, exercise, and eating, and how to encourage and communicate with active individuals without commenting on weight, shape, and food in negative ways. The third and final part focuses on how to reach out to and talk to active individuals (henceforth referred to as “clients”) whose behavior raises concerns, and how to guide individuals in need of professional help. I Care is interactive, with exercises and quizzes for the participants to engage in. Most educational content is conveyed via brief videos, but participants also follow three fictitious cases with different profiles throughout the program. Going through I Care takes up to 3 h, but participants can pause and come back later.

### Procedure

Eligible participants interested in taking part were invited to contact the first author via e-mail. Participants were then provided with a free I Care account. When the participant first logged in, information about the study and an online consent form appeared in a pop-up window. After consent, the participant commenced to answer the PRE survey in the same pop-up window. After completion, the I Care program launched. After concluding I Care, the POST survey appeared in a new pop-up window. The pre- and post-survey were created using Survey Monkey. Participants were identified, and their PRE and POST measures were matched using the four last digits of their personal identification number. Ethical approval was sought but judged by the ethical review authority not to be needed, as no intervention or sensitive data that falls within the remit of research ethics legislation was included in the study (Swedish Ethical Review Authority reg. no. 2020–04,612).

### Instruments

The survey started with demographic information (age, gender, sports organization, role, geographic location), a question about whether participants were aware of documented policies/action plans concerning ED within the organization (variable called “Aware_Policy” below), and whether the participant knew how to access it (“Access_Policy”).

After this, the participants completed the compulsive exercise test (CET; [19]), a 24-item instrument scored 0–5 (never true-always true) measuring attitudes and behaviors in relation to physical activity. The CET is summarized in a total scale score and five subscale scores measuring Avoidance and rule-driven behavior (present Cronbach’s alpha PRE = 0.876; POST = 0.899), Weight control exercise (alpha PRE = 0.768; POST = 0.748), Mood improvement (alpha PRE = 0.630; POST = 0.887), Lack of exercise enjoyment (alpha PRE = 0.860; POST = 0.857), and Exercise rigidity (alpha PRE = 0.528; POST = 0.649). Since internal consistencies were low for the Mood improvement and Exercise rigidity subscales, we did not analyze these data further. After completing the CET in relation to themselves, the participants completed the CET again from the perspective of the clients (these data will not be treated further here). The CET has shown satisfactory psychometric properties [19–21].

After the CET, a survey intended to briefly capture the focus areas of I Care followed (henceforth "I Care evaluation items”), with items measuring knowledge and awareness about EDs, healthy leadership, and ability to reach out to and guide athletes for whom there are concerns. The survey was developed in several steps. First, five independent ED experts with good background knowledge of the field and of the I Care intervention generated a large number of relevant questions. After discussion, the group agreed upon 34 items, all formulated as statements rated on a 1–7-point Likert scale (from “Not at all true” to “Very true”). These items were piloted in a group of over 30 coaches receiving “We Care”, a similar education to I Care but delivered face-to face. Studying the pilot data, 10 items showed minimal or no variance and were thus excluded. 24 items remained and were used in the present study. Examples of items are: “You can tell if a person has an eating disorder just by looking at him/her”, “To me, exercise is a way of changing the shape of my body” and “I know how to talk to a person who exercises compulsively”. Survey items are presented in Additional file 1: Table S1.

### Statistical analysis

Analyses were performed using IBM SPSS 26 for Mac. To avoid Type I error, all *p*-values were subjected to false discovery rate (FDR) correction [22] within each block of outcome variable comparisons (CET; I Care evaluation variables). Attrition analyses (comparing those who responded to both PRE and POST surveys to those who only responded to PRE) were performed on baseline data using χ^2^ and *t*-tests, or Welch’s *t* where Levene’s test for equality of variances was significant. The I Care evaluation items were reduced using principal components analysis (PCA) with varimax rotation on baseline data, including the Kaiser–Meyer–Olkin (KMO) test of sampling adequacy (requiring a value > 0.60 for adequacy; [23]) and Bartlett’s test of sphericity, and parallel analysis was run to decide the number of components along with inspecting the scree plot. Missing values were replaced with the mean, and > 0.50 was required to load on a component, along with < 0.40 on any other component. No items cross-loaded according to these criteria. PRE and POST scores were then calculated as means of the items in each component, and internal consistency for the components was investigated using Cronbach’s alpha.

Component scores and CET subscales were compared pre-to-post using paired *t*-tests or nonparametric Sign tests where departures from normality were detected (skewness or kurtosis >  ± 1). The possible covariate age was not included since it did not show FDR-corrected significant association with any outcome variable. Gender differences were tested using independent *t*-tests/Welch’s *t*. Repeated measures ANOVA found no significant gender by time interactions in any outcome (data not shown). Effect sizes are shown as Cramer’s *V* for χ^2^ tests, Cohen’s *d* for independent *t*-test comparisons, and *d*_z_ for paired *t*-tests (using G*Power 3.1.9.6 for Mac), all with interpretation conventions small > 0.20, moderate > 0.50, and large > 0.80, and *r* for Sign tests, interpreted as small > 0.10, moderate > 0.30, and large > 0.50.

## Results

### Attrition and gender differences

Comparing those who completed both pre- and post-surveys to those who only responded to pre, we found no FDR-corrected significant differences in age, gender distribution, any of the CET subscales, or any I Care evaluation item. No baseline gender differences were observed on any outcome except on Weight/Body/Diet Talk (see below), where men scored higher (*M* = 3.93, *SD* = 1.253) than women (*M* = 3.01, *SD* = 1.340; *t* = -3.37, *p* < 0.001, *d* = 0.71).

### PCA of I Care evaluation items

In the I Care evaluation item PCA, KMO was 0.747 and Bartlett’s *p* was < 0.001, suggesting appropriateness of the data. Both the scree plot and parallel analysis suggested five components, explaining 64.33% of the variance (Eigenvalues ≥ 1.36). Items in each component along with loadings are shown in Additional file 1: Table S1. The first component concerned confidence and perception of knowledge about how to recognize problematic eating and exercise behavior and how to approach and refer clients concerning such issues (Confidence & Knowledge; PRE Cronbach’s α = 0.921; POST α = 0.885). The second component included items about organizational awareness and communication around diet and exercise-related risk for ED and general health (Organizational Awareness, PRE α = 0.824; POST α = 0.774). The third component concerned whether questionable messages were conveyed within the organization to clients regarding weight, calorie compensation, and deserving food (Negative Messaging, PRE α = 0.831; POST α = 0.889). The fourth component concerned whether the participant communicated with clients about recuperation, stress, exercise technique, and whether the participant endorsed the organization’s messages concerning exercise (Healthy Communication, PRE α = 0.637; POSTα = 0.598), and the fifth and final component concerned the degree to which participants talked to clients about diet, weight, and body appearance (Weight/Body/Diet Talk, PRE α = 0.733; POST α = 0.747). The item asking about participants’ ability to recognize whether a person had an ED (Recognize ED) did not load on any component and was therefore analyzed separately.

### PRE to POST comparisons

Table 1 shows descriptive statistics and comparisons for CET and I Care evaluation components and the Recognize ED variable before and after I Care. We observed significant decreases in CET subscales Avoidance and rule-driven behavior (small effect) and Lack of exercise enjoyment (large effect). For the I Care evaluation components, Organizational Awareness and Healthy communication did not change significantly, but the other components did so in the intended directions: a large-effect increase in Confidence & Knowledge, a small decrease in Negative Messaging, and a moderate decrease in Weight/Body/Diet Talk. Further, the Recognize ED item decreased significantly with a large effect.

**Table 1:** Descriptive statistics, paired *t*-tests or Sign tests where non-normal distribution was found, *d*_z_ or *r* effect sizes, and FDR corrected significance for each outcome variable

Variable	PRE *M* (*SD*)	POST *M* (*SD*)	*t* / *Z*	*df*	*p*	*d*_z_	FDR S/ns
CET avoidance and rule-driven behavior	1.82 (.980)	1.64 (.917)	3.989^1^	148	< .001	.33^2^	S
CET weight control exercise	1.80 (.937)	1.71 (.844)	2.026	149	.045	.17	ns
CET lack of exercise enjoyment	2.40 (.905)	.84 (.767)	8.969^1^	148	< .001	.73^2^	S
Confidence and knowledge	4.09 (1.419)	5.26 (1.016)	− 12.059	138	< .001	1.02	S
Organizational awareness	4.70 (1.593)	4.77 (1.722)	− .794	138	.428	.07	ns
Negative messaging	2.40 (1.335)	2.25 (1.380)	2.409^1^	138	.016	.20^2^	S
Healthy communication	5.59 (1.021)	5.50 (.993)	.603^1^	138	.546	.05^2^	ns
Weight/Body/Diet Talk	3.23 (1.380)	2.77 (1.313)	4.720^1^	138	< .001	.39^2^	S
Recognize ED	3.09 (1.560)	2.14 (1.412)	6.526^1^	138	< .001	.53^2^	S

FDR = false discovery rate; CET = Compulsive Exercise Test; S = Significant; ns = not significant; ED = eating disorder

^1^Sign test *Z* statistic is shown

^2^*r* effect size, calculated as *r* = Z/$\sqrt{N}$

Concerning Aware_Policy and Access_Policy, results showed significant changes from PRE to POST (see Table 2). The proportion who responded Yes to each question increased (31% to 47% and 33% to 51%, respectively), and Don’t know-responses decreased (48% to 35% and 55% to 39%, respectively). The effect was primarily due to about a third of PRE Don’t know-responses having been changed to Yes at POST.

**Table 2:** Pre-and post distribution, χ^2^ and *V* effect size for the policy/action plan items

Item		Yes	No	Don’t know	Total
Are there guiding documents (policy and action plan) related to ED? χ^2^(4) = 146.017 *p* < .001 *V* = .698	Yes	45 (96%)	0	2 (4%)	47 (31%)
	No	2 (7%)	23 (74%)	6 (19%)	31 (21%)
	Don’t know	24 (33%)	2 (3%)	46 (63%)	72 (48%)
	Total	71 (47%)	25 (17%)	54 (36%)	150 (100%)
If yes, do you have access to such documents? χ^2^(4) = 130.660 *p* < .001 *V* = .726	Yes	39 (95%)	0	2 (5%)	41 (33%)
	No	1 (7%)	12 (80%)	2 (13%)	15 (12%)
	Don’t know	23 (34%)	1 (2%)	44 (65%)	68 (55%)
	Total	63 (51%)	13 (11%)	48 (39%)	124 (100%)

## Discussion

The purpose of the present study was to investigate the effects of the online intervention I Care on sport coaches´ attitudes, communication, and perception of knowledge about EDs. Results indicate that after completing I Care, coaches’ attitudes about body shape, weight, and exercise had improved significantly and they were much less prone to talk about for instance diet and weight in problematic ways. Further, coaches’ sense of possessing knowledge about CE and EDs in general, as well as about where to refer individuals with problematic behaviors had improved at follow-up. They also reported increased confidence in both recognizing warning signs of EDs and in approaching individuals struggling with EDs and CE. Finally, organizational awareness, and the experience of negative messaging and healthy communication within one’s organization did not change to any notable extent (negative messaging was significant, but the effect size was very small). The results are overall consistent with the course intentions and content and suggest that a brief online intervention like I Care may potentially both successfully educate about EDs and CE, and boost coaches’ confidence and readiness to act when they meet individuals at risk in their coaching role. This is an important finding since knowledge about EDs among coaches may lead to earlier detection and possibility for intervening with problematic behaviors, which in turn may contribute to better prognosis for individuals at risk [16].

### Attitude change

Interestingly, participants’ scores on the CET subscales “Lack of exercise enjoyment” and “Avoidance and rule-driven behavior” decreased significantly from pre to post measure. Since most individuals completed I Care over the course of a day, these changes most likely indicate a renewed attitude toward one’s own behaviors rather than actual behavior change. Both the CET items and the I Care course may have reminded participants of exercise being a positive choice rather than a habit or duty. The lack of a control group makes conclusions difficult to draw, but the possibility of such a positive effect on coaches’ self-perception is intriguing and could be studied in future research. Briefly, there are unfortunately no studies investigating test–retest properties of the CET, but in a similar measure of compulsive exercise in ED there were no mean score changes from test to retest [24], possibly suggesting that responding to a psychometric measure alone would not cause a change in scores.

A recent review [25] suggested that athletes often perceive their coaches as influential in relation to eating pathology via for instance pressure and comments about body weight, shape, size, and appearance, as well as diet prescription and advice. Hence, increasing awareness about this negative influence and how to avoid it is key. At POST participants reported being significantly less prone to talk about diet, weight, and body appearance with their clients, suggesting incorporation of the newfound knowledge; gaining important insights about their own behavior and deciding to change. Thus, the lower scores at post may be most accurately interpreted as a “declaration of intent”, rather than an actual behavior change since this was unlikely to have had time to occur. According to both theory and research, changed attitudes and the formation of intentions are key components precipitating behavior change [26]. Further, dissonance-based interventions for behavior change have found considerable support, especially perhaps the “hypocrisy paradigm”, where people are made aware of their own past transgressions against some desired norm or behavior [27], which is reminiscent of what may indirectly have happened here.

### Perceived knowledge change

Participants felt more knowledgeable about EDs and CE post I Care, something that is also suggested by the decrease in scored ability to recognize EDs. A common misperception is that you can tell if an individual suffers from an ED just by looking at them. In I Care, a primary focus is to bust myths like these and nuance the way participants think about EDs, for instance by highlighting (e.g., with the fictive cases) how EDs may be expressed in various ways. The fact that participants felt more confident in recognizing warning signs hopefully indicates an increased knowledge about the variety of symptoms and behaviors to be wary of, rather than just focusing on bodily appearance. However, despite the warning signs, there is no way of knowing what is going on for someone unless you ask them. Therefore, it is of particular importance that coaches feel able and confident in talking to individuals they have concerns about. I Care seems to increase coaches’ confidence in approaching individuals they worry about and equipping them in ways so that they are better able to refer individuals that indeed have problems. Other efforts have demonstrated similar positive results when intervening with coaches: increased knowledge of ED [17] as well as behavior change [28]. However, previous efforts have often been rather rigorous and time-consuming. An advantage with I Care is the fact that it is online and brief, making it accessible, cost- and time-effective, and not very resource-intense.

### Organizational awareness and communication

Organizational awareness did not improve significantly and although negative messaging and healthy communication within the organization changed slightly from pre to post. Mean scores suggest that participants already perceived attitudes and communications within their organizational contexts as positive, leaving marginal room for improvement. Also, these items measured factors outside participants’ control, and thus were less likely to change.

### Limitations

A key limitation of the present study is the lack of a control group. Thus, no firm inferences can be drawn about the efficacy of the I Care course, although findings are suggestive and consistent with the course intentions and content. Further, we do not know if participants’ reported attitude/behavior changes led to actual change in communication, management, or detection of ED, as we had no follow-up data or criterion measures such as actual referrals or pre-post measures of clients’ perceptions or experiences of the coaches’ behavior. We also had no long-term follow-up data to measure potential long-term effects on coaches’ attitudes and behaviors. Such measures would be useful to include in future research.

A possible source of bias in responses might be that coaches working in this field know what responses are expected from them, especially in view of the organizations apparently having positive communication policies in place. Although participants were ensured that survey scores were anonymous and would not be communicated at individual level to anyone but the researchers, there may have existed pressure to report in a socially/organizationally desirable way, and our results may thus be overestimates of actual changes. This further underscores the previous point raised, and future studies should aim towards using other metrics preferably measuring actual behavioral change, to evaluate the effects of the intervention.

Further, we had a fair amount of missing data on Access_Policy, probably because there was no automatic skip rule after Aware_Policy; the question of whether participants had access to an organizational policy would have made little sense to those who responded “Don’t know” to whether they at all knew about any such policy. Also, Cronbach’s alpha was poor for Healthy Communication, and results should be interpreted with caution.

### Conclusions

We found positive post effects of I Care regarding perceived knowledge about CE and EDs, improved attitudes about one’s own exercise behavior, “declared intent” to avoid focusing on diet, body appearance and weight in communication with clients, and greater confidence in approaching and referring clients at risk. These findings are encouraging, since greater knowledge and awareness among coaches may lead to earlier detection and intervention which can contribute to better outcomes for the athlete, and especially also in the light of the brevity, accessibility, and cost-effectiveness of I Care. Potential long-term effects for both coaches and their clients will need further investigation, but these initial results are promising.

## Supplementary Information

**Additional file 1**. I Care evaluation items.

## Data Availability

The datasets generated and/or analysed during the current study are available in the [NAME] repository, [PERSISTENT WEB LINK TO DATASETS] Data will be uploaded prior to publication.

## Abbreviations

ED: Eating disorder
CE: Compulsive exercise
IOC: International Olympic Committee
ACSM: American College of Sports Medicine
CET: Compulsive exercise test
