# Supplementary material for: Preliminary evaluation of the online course “I Care” targeting eating disorder knowledge and attitudes among sports coaches and fitness instructors
Source: J Eat Disord. 2022 Sep 29;10:142. doi: 10.1186/s40337-022-00663-1 (PMC9520860; doi:10.1186/s40337-022-00663-1)
Supplement: Supplementary file 1 — Additional file 1. I Care evaluation items. [file 40337_2022_663_MOESM1_ESM.docx]

**Supplementary Table 1.** I Care evaluation items, including rotated component matrix PCA loadings for each item.

| **Items** | Confidence & Knowledge | Organizational Awareness | Negative Messaging | Healthy Communication | Weight/Body/Diet Talk |
| --- | --- | --- | --- | --- | --- |
| **I know how I could talk to a person who exercises in an excessive way** | .859 |  |  |  |  |
| **I know how I could talk to a person who has problems with their eating** | .838 |  |  |  |  |
| **I know where I can refer a person who exercises in an excessive way** | .816 |  |  |  |  |
| **I feel confident about how to approach a person who has problems with their eating** | .781 |  |  |  |  |
| **If someone has problems with excessive exercise, I know how to handle that within my organization** | .775 |  |  |  |  |
| **I know where I can refer a person who has problems with their eating** | .773 |  |  |  |  |
| **I feel confident about how to approach a person who exercises in an excessive way** | .769 |  |  |  |  |
| **I would know if someone exercises excessively** | .769 |  |  |  |  |
| **If someone has problems with their eating, I know how to handle that within my organization** | .748 |  |  |  |  |
| **I would know if someone has problems with their eating** | .567 |  |  |  |  |
| **Within my organization, we talk about risk of eating disorders related to excessive exercise** |  | .833 |  |  |  |
| **Within my organization, we talk about health risks related to excessive exercise** |  | .826 |  |  |  |
| **My organization conveys healthy messages about diet** |  | .576 |  |  |  |
| **How often are comments like these used within your organization?:** |  | | | | |
| Comments about compensation or “exercising away” something eating, e.g., “Push harder and burn more calories” |  |  | .874 |  |  |
| Comments about deserving to eat, e.g., “Now you’ve worked out hard and deserve some comfort food” |  |  | .821 |  |  |
| Comments about weight loss, e.g., ”Push harder, think about the beach this summer!” |  |  | .806 |  |  |
| **I talk about rest with clients** |  |  |  | .707 |  |
| **I talk about stress with clients** |  |  |  | .633 |  |
| **I talk about exercise technique with clients** |  |  |  | .595 |  |
| **I fully support my organization’s messages about exercise** |  |  |  | .505 |  |
| **I talk about physical appearance with clients** |  |  |  |  | .822 |
| **I talk about weight with clients** |  |  |  |  | .805 |
| **I talk about diet with clients** |  |  |  |  | .550 |
